# Sex and Socioeconomic Differentials in Child Health in Rural Bangladesh: Findings from a Baseline Survey for Evaluating Integrated Management of Childhood Illness

**Published:** 2008-03

**Authors:** Shams El Arifeen, Abdullah H. Baqui, Cesar G. Victora, Robert E. Black, Jennifer Bryce, D.M.E. Hoque, E.K. Chowdhury, N. Begum, T. Akter, A. Siddik

**Affiliations:** 1 ICDDR,B, GPO Box 128, Dhaka 1000, Bangladesh; 2 University of Pelotas, Pelotas, Brazil; 3 Johns Hopkins Bloomberg School of Public Health, 615 North Wolfe Street, Baltimore, MD 21205, USA

**Keywords:** Child care, Child health, Hospitalizations, Integrated Management of Childhood Illness, Morbidity, Socioeconomic status, Bangladesh

## Abstract

This paper reports on a population-based sample survey of 2,289 children aged less than five years (under-five children) conducted in 2000 as a baseline for the Bangladesh component of the Multi-country Evaluation (MCE) of the Integrated Management of Childhood Illness strategy. Of interest were rates and differentials by sex and socioeconomic status for three aspects of child health in rural Bangladesh: morbidity and hospitalizations, including severity of illness; care-seeking for childhood illness; and home-care for illness. The survey was carried out among a population of about 380,000 in Matlab upazila (subdistrict). Generic MCE Household Survey tools were adapted, translated, and pretested. Trained interviewers conducted the survey in the study areas. In total, 2,289 under-five children were included in the survey. Results showed a very high prevalence of illness among Bangladeshi children, with over two-thirds reported to have had at least one illness during the two weeks preceding the survey. Most sick children in this population had multiple symptoms, suggesting that the use of the IMCI clinical guidelines will lead to improved quality of care. Contrary to expectations, there were no significant differences in the prevalence of illness either by sex or by socioeconomic status. About one-third of the children with a reported illness did not receive any care outside the home. Of those for whom outside care was sought, 42% were taken to a village doctor. Only 8% were taken to an appropriate provider, i.e. a health facility, a hospital, a doctor, a paramedic, or a community-based health worker. Poorer children than less-poor children were less likely to be taken to an appropriate healthcare provider. The findings indicated that children with severe illness in the least poor households were three times more likely to seek care from a trained provider than children in the poorest households. Any evidence of gender inequities in child healthcare, either in terms of prevalence of illness or care-seeking patterns, was not found. Care-seeking patterns were associated with the perceived severity of illness, the presence of danger signs, and the duration and number of symptoms. The results highlight the challenges that will need to be addressed as IMCI is implemented in health facilities and extended to address key family and community practices, including extremely low rates of use of the formal health sector for the management of sick children. Child health planners and researchers must find ways to address the apparent population preference for untrained and traditional providers which is determined by various factors, including the actual and perceived quality of care, and the differentials in care-seeking practices that discriminate against the poorest households.

## INTRODUCTION

Bangladesh has witnessed remarkable declines in infant and child mortality in the last two decades. In the early 1980s, rates of mortality of children aged less than five years (under-five children) were as high as 200 per 1,000 livebirths. By 2000, the rate had declined over 50% to 94% per 1,000 livebirths (1). However, the most recent demographic and health survey (DHS) in Bangladesh (BDHS 1999–2000) indicates that the rate of decline is slowing (2-4). A similar trend has been observed in the Matlab field site of the International Centre for Diarrhoeal Disease Research, Bangladesh (ICDDR,B), a rural area where ICDDR,B has been maintaining a longitudinal health and demographic surveillance system (HDSS) for almost 40 years (5). Data from these sources showed that much of the decline in child mortality in Bangladesh in the last 10 years has been in rural areas. Rates in urban areas have changed very little. This suggests that routine child health services in the public sector may be reaching their peak impact, and further improvements in child survival may require substantial changes in care-seeking patterns, in how child healthcare services are delivered, the range of services, and in the channels used for reaching different segments of the population. Despite considerable investment in public-health infrastructure, public-health services in Bangladesh are characterized by poor quality and low rates of use (6-8).

Inequities in the use of child health services and health status exist in many parts of the world (9).

Results of analyses of the BDHS 1996–1997 by the Health, Nutrition and Population Programme of the World Bank showed significant differentials in care-seeking by wealth (10). For example, children with pneumonia in the least poor 20% of families were more than twice as likely to be taken to a medical facility for care (51%) than children with pneumonia in the poorest 20% of families (23%). Of children in the poorest 20% of families, boys with pneumonia were 61% more likely to be taken for medical care than girls. Even in the least poor 20% of families, boys with pneumonia were 45% more likely to receive medical care (10).

The Integrated Management of Childhood Illness (IMCI) strategy brings together previous vertical programmes for the management of childhood illnesses. It encompasses skilled health workers in the first-level facilities, functioning, and supportive health systems that include supervision, adequate and sustained supply of drugs, effective referral systems, and engagement of families and communities in improving key practices (11). The strategy is designed to be adapted to local country settings. The IMCI strategy, if well-adapted and implemented, has the essential attributes to address many problems with child health services as currently provided.

The Government of Bangladesh officially adopted the IMCI strategy in 1998; however, it was only in late 2001 that pilot implementation began in two subdistricts. One of these subdistricts—Matlab—is also one of five global sites for the Multi-country Evaluation (MCE) of IMCI Effectiveness, Cost and Impact (12). The Bangladesh component of the MCE study is using an experimental design to assess the impact of IMCI on mortality and other indicators. This report presents results from the baseline household survey conducted for this study in 2000. It examines rates and differentials by sex and socioeconomic status for three aspects of child health in rural Bangladesh: morbidity and hospitalizations, including severity of illness; care-seeking for child illness; and home-care for illness.

## MATERIALS AND METHODS

### Study site

The study was conducted in Matlab upazila (subdistrict) of Bangladesh, which has a population of about 500,000. ICDDR,B provides direct child and reproductive health services to approximately 120,000 persons in Matlab. The remaining portion (~380,000) of the Matlab population is served by the health and family-planning programme of the Government of Bangladesh (GoB) and was the primary study area for this project.

### Study sample

A demographic survey, based on a complete census of the study area, provided the sample for a household health and morbidity survey. The field work was carried out over six months from April to October 2000. The incidence of both diarrhoea and pneumonia varies seasonally in the survey area as in other parts of Bangladesh, and the survey period included a peak diarrhoea season. A sample of 2,200 under-five children was required for the survey to explain post-intervention changes of at least 20% in key family practices relating to child health between the IMCI and the comparison area with a power of 80%. Based on the initial numbers of under-five children identified in the demographic survey, we estimated that we would obtain the required number of children if we took a systematic sample of every 16th child found in the demographic survey. This sampling scheme also ensured that multiple children were not selected from the same household.

### Indicators

The survey questionnaire collected information from the caretaker of the child on healthcare-seeking practices, illness in the previous two weeks, and care-seeking for those illness episodes. Data were also collected on home-care for illness and compliance with healthcare advice. Weight and height/length of the children were measured using the Uniscales (UNICEF, Copenhagen) and locally-produced length/height boards. A generic questionnaire, developed by the World Health Organization/MCE team (http://www.who.int/imci-mce/Methods/household_survey.htm), was adapted for use in Bangladesh. The Principal Investigator translated it into Bangla, and it was then pretested for use in Matlab. The different sections were colour-coded to facilitate their use.

### Procedures

Seven experienced female interviewers were trained in the use of the questionnaire, interviewing techniques, and anthropometric measurements and on the use of village sketch maps and household lists to locate sampled children. The median interview time was 60 minutes.

### Analysis

The analysis was based on the standard MCE indicators and equity analysis guidelines (http://www.who.int/imci-mce/) adapted for use in Bangladesh. Specific illnesses investigated in this analysis included reported fever, diarrhoea, and probable pneumonia (defined as cough with rapid or difficult breathing which was not due to a blocked nose). The differences in illness and care-seeking rates were tested using the chi-square tests of independence/homogeneity. The exact tests were performed if group frequencies were small. We investigated about how rates of illness and care-seeking varied among those with different demographic, household, and illness characteristics. We also assessed associations of these variables for hospitalization rates, home management of illness and compliance with provider advice. Compliance was defined as adherence to the advice of the provider about follow-up, referral, and treatment of the child.

**Fig. 1:**
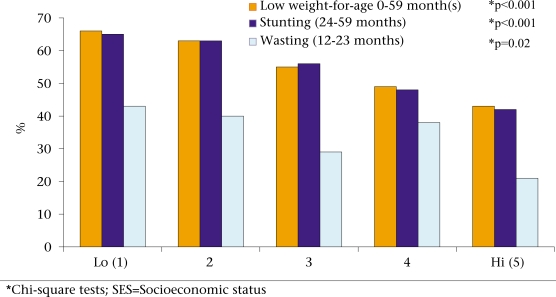
Low weight-for-age, stunting, and wasting rates by SES quintiles

An index of socioeconomic status was developed using methods pioneered by Gwatkin and his colleagues (10). A standardized factor score derived from principal component analysis was assigned to each of 13 household assets or possessions which included beds, watch, chairs/tables, cabinets, radio, television (TV), bicycle, boat, cows/goats, chickens/ducks, ownership of homestead land, and structural materials used in the house. These possessions are commonly used in Bangladesh for economic assessments and considered to be discriminatory (10). The index was calculated from the weighted sum of these household assets and possessions, the weight being the score for each indicator. The principal component analysis gave the highest score (weight) to households owning 1–2 goat(s) and the most negative score to landless households. The variance of the first principal component (eigenvalue) was 3.9 which explained 15% of the total variance. The second principal component, uncorrelated with the first principal component, explained 8% of the remaining variation, and the third principal component, uncorrelated with previous principal components, explained 7% of the remaining variation. The external validity of the asset index was assessed by examining associations between index scores and other indicators with known levels of association with socioeconomic status. Figure 1 presents rates of low weight-for-age, stunting, and wasting by the socioeconomic status quintiles. We observed lower rates of poor nutritional status with increasing socioeconomic status. This reflects positively on the validity of this index.

All analyses were carried out using the Stata software (version 7).

## RESULTS

In total, 2,289 under-five children were sampled; of them, interviews were completed for 2,066 (90%). Five children had died since the demographic survey. The families of 142 children were not found at the time of the follow-up survey. The 142 lost-to-follow-up children did not differ from the 2,066 for whom interviews were completed in terms of wealth categories, but were more likely to be girls (63%) than the completed sample (48%); 69 children were no longer aged less than 60 months; and interviews were incomplete for seven children. The remainder of this analysis is based on the 2,066 children for whom interviews were completed.

Characteristics of the sample of children are summarized in Table 1. Fifty-two percent (n=1,076) of the children were boys. There is some under-representation at the younger ages relative to the distributions reported from other sample surveys (2). This bias can be explained by the delay of 1–2 month(s) between the demographic and the morbidity survey, during which time the youngest children became slightly older.

**Table 1:** Two-week prevalence of childhood illnesses (rate per 100 children) and distribution of hospital admissions in the year preceding the survey, by selected child, household, and illness characteristics

Sociodemographic characteristics	No. of children	Any illness*	Fever*	Diarrhoea*	Probable pneumonia*	No. of hospitalizations (rate/child-year)†	
All children	2,066	64.7 (1,336)	45.2 (934)	13.1 (270)	4.2 (86)	114	(0.055)
Gender							
Boys	1076	66.4	45.9	13.8	4.7	73	(0.068)
Girls	990	62.8	44.4	12.3	3.6	41	(0.041)
		p=0.09	p=0.50	p=0.33	p=0.25	p=0.01	
Age (months) of children							
0–5	99	71.7	48.8	12.1	3.0	0	(0.000)
6–11	265	72.8	52.1	13.2	3.4	10	(0.038)
12–23	434	68.9	50.5	18.4	5.3	44	(0.101)
24–35	452	66.2	47.6	12.6	4.2	32	(0.071)
36–47	434	63.1	41.9	10.8	5.1	17	(0.039)
48–59	382	52.4	34.6	10.2	2.4	11	(0.029)
		p<0.01	p<0.01	p<0.01	p=0.32	p<0.01	
Socioeconomic status							
Lo (1)	434	64.8	46.8	15.0	3.9	21	(0.048)
2	412	62.4	45.2	12.4	2.9	19	(0.046)
3	406	62.8	45.1	11.6	4.7	22	(0.054)
4	413	68.3	46.7	13.1	4.1	26	(0.063)
Hi (5)	401	65.1	42.1	13.2	5.2	26	(0.065)
		p=0.41	p=0.67	p=0.67	p=0.54	p=0.74	

*Two-tailed p values based on chi-square tests of independence/homogeneity

†Two-tailed p values based on chi-square tests of goodness-of-fit; Lo=Low; Hi-High

### Morbidity patterns and reported hospitalizations

Table 1 provides a summary of the two-week prevalence of common childhood illnesses in this sample and the distribution of reported hospital admissions in the year preceding the survey, by gender, age, and the socioeconomic status quintile of the child. Almost two-thirds of the children reported at least one illness in the two weeks preceding the survey. About 45% had a fever, about 13% had diarrhoea, and about 4% had probable pneumonia. Boys were somewhat more likely than girls to have an illness reported for the recall period, although this finding was not statistically significant. Any illness, fever, and diarrhoea were common at younger ages, but probable pneumonia did not differ with the age of the child. We did not find differences in rates of reported morbidity by socioeconomic status.

There were 114 hospitalizations in the previous year among the sampled children (Table 1). Of the 97 hospitalized children, 82 were hospitalized only once, 13 twice, and only two thrice—all for pneumonia. About two-thirds of the hospitalizations were due to diarrhoea, and about 25% for pneumonia (data not shown). There were more hospitalizations among boys than among girls and among 12–35 months old children than among either younger or older children (Table 1). Although children from the upper socioeconomic status quintiles were more likely to be hospitalized than those in the lower quintiles, the distribution of hospitalizations did not differ significantly by socioeconomic status.

**Fig. 2:**
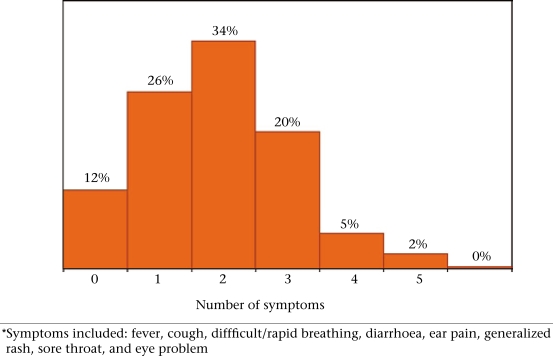
Distribution of children by number of symptoms* (selected) reported in previous 2 weeks

**Fig. 3:**
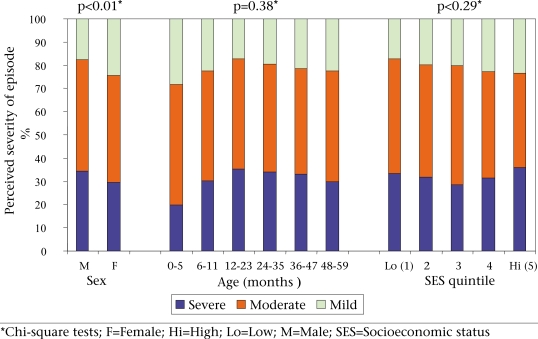
Perceived severity of illness episode by sex and age of children

Figure 2 shows the distribution of the 1,336 children who were ill in the two weeks preceding the survey by the total number of reported symptoms. We limited this analysis to eight key symptoms. About 12% of the children did not have any of the eight symptoms (i.e. they had some other illnesses), and about a quarter had only one symptom. The majority of these children, however, reported more than one symptom. Figure 3 presents results relating to judgments of caretakers about the severity of illness of their children by sex and age of the child and the socioeconomic status quintile of the fami-ly. Boys were significantly more likely than girls to have severe episodes reported by the caretaker, but no significant differences were found by age of the child or by wealth.

### Care-seeking practices

Few of the ill children were reported to have been taken for care to a trained health provider (Table 2). No care from outside the home was sought for about one-third of the children with a reported illness, and among those for whom care was sought, high proportions were taken to a village doctor, which includes untrained village practitioners and sellers of modern drugs. Approximately one in 10 ill children was taken to a traditional provider, a varied group that includes herbalists and herbal drug-sellers, homeopaths, traditional healers, and traditional midwives. Only 8% of the children were taken to a trained health provider, defined as a health facility (4%), a trained medical doctor (3%), or a trained community health worker (1%). Of the ill children for whom care was sought outside the home, 13% reported taking their children to more than one provider with an average of 1.18 providers per illness episode.

**Table 2:** Percentage of sick children seeking care by source of care, number of providers, and by selected child, household, and illness characteristics, Matlab, Bangladesh, 2000

Sociodemographic and illness characteristics	No. of sick children	No care sought for illness	Care sought for illness					Among those seeking care (n=849)	
			Any care*	Trained†	Village doctor‡	Traditional	Other	% seeking care from >1 providers	Mean no. of providers¶
All children	1,336	36.5	63.5	7.6	41.8	10.7	3.4	13.3	1.18
Gender									
Boys	714	36.0	64.0	7.3	43.4	10.6	2.7	15.7	1.21
Girls	622	37.0	63.0	8.0	39.9	10.8	4.3	10.5	1.15
Age (months) of children									
0–5	71	36.6	63.4	9.9	25.4**	23.9	4.2	24.4	1.36††
6–11	193	32.6	67.4	10.4	38.3	15.0	3.7	15.4	1.22
12–23	299	36.1	63.9	8.4	43.1	10.0	2.4	16.8	1.24
24–35	299	35.8	64.2	5.7	45.8	9.4	3.3	14.6	1.19
36–47	274	35.8	64.2	5.8	44.9	8.8	4.7	5.1	1.07
48–59	200	42.5	57.5	8.5	38.5	7.5	3.0	11.3	1.14
Socioeconomic status									
Lo (1)	281	43.4	56.6††	3.9‡‡	40.2	9.3	3.2	9.4	1.13
2	257	39.7	60.3	4.3	39.3	15.2	1.6	15.5	1.20
3	255	34.9	65.1	7.8	43.5	11.0	2.8	16.3	1.20
4	282	35.8	64.2	8.2	41.5	9.9	4.6	11.1	1.17
Hi (5)	261	28.0	72.0	14.2	44.4	8.4	5.0	14.4	1.21
Perceived severity									
Mild	276	57.6	42.4‡‡	2.9‡‡	30.1‡‡	7.6	1.8	5.1	1.06**
Moderate	629	36.4	63.6	5.9	43.6	11.0	3.1	13.5	1.18
Severe	431	23.0	77.0	13.2	46.6	12.3	4.9	16.0	1.23
At least 1 danger sign present									
Yes	362	27.4	72.7‡‡	11.1††	48.3††	10.8	2.4	16.0	1.25**
No	974	39.8	60.2	6.4	39.3	10.7	3.8	12.1	1.15
Number of symptoms									
0	156	38.4	61.6‡‡	6.4††	39.7††	10.3	5.1	11.5	1.20
1	353	45.8	54.2	5.3	33.4	11.1	4.3	10.5	1.14
2	454	36.5	63.5	7.4	44.9	9.3	1.8	13.5	1.16
3	273	30.0	70.0	7.6	46.8	12.4	2.9	13.6	1.20
4 +	100	17.0	83.0	18.0	46.0	12.0	7.0	20.5	1.33
Duration (days) of illness§									
0–6	756	47.0	53.0‡‡	4.5‡‡	36.6‡‡	9.8	2.0	8.0	1.09‡‡
7–13	300	25.7	74.3	8.3	51.3	13.0	1.7	14.4	1.17
14 +	280	19.3	80.7	15.4	45.4	10.7	9.3	21.7	1.36
Type of illness									
Fever	934	33.8	66.2††	8.1	44.8††	10.8	2.5	14.4	1.19
Diarrhoea	270	34.1	65.9	5.6	49.6††	8.2	2.6	17.4	1.25
Probable pneumonia	86	32.6	67.4	7.0	44.2	12.8	3.5	19.0	1.29

*p value compares ‘any care’ with ‘no care’, and based on chi-square tests of independence/homogeneity

†p value compares ‘care from trained provider’ with ‘no/other care’, and based on chi-square tests of independence/homogeneity

‡p value compares ‘care from village doctor’ with ‘no/other care’, and based on chi-square tests of independence/homogeneity

¶p values based on *t*-test and ANOVA test

§Many illness episodes were censored (still ongoing)

**p<0.05;

††p<0.01;

‡‡p<0.001;Lo=Low; Hi=High

We investigated possible associations between characteristics of the child and illness and care-seeking practices (Table 2). Care-seeking practices were similar in girls and boys. The significant differences were found in reported care-seeking practices by the socioeconomic status of the family. For example, 72% of children from the upper socioeconomic status quintile were taken outside the home for care compared to 57% among those from the lowest quintile. The overall rate for seeking care outside the home was also higher when the disease was perceived as being severe and when danger signs were present and was directly associated with the number of symptoms, duration of the episode, and type of illness.

In households of higher socioeconomic status or if the illness was perceived to be severe, if there was a danger sign present, if several symptoms were present at the same time, or if duration of the illness was long, caretakers of the sick child were more likely to go to a trained provider for care (Table 2). No difference was found by age of the child or by the type of illness, but care from a trained provider was most often sought when the child had respiratory distress or rapid breathing (data not shown).

The use of village doctors, traditional care providers, and trained providers increased with the severity of illness, but even when the caretaker perceived illnesses as severe, only 13% of the children were taken to a trained provider, and 23% did not seek any care outside the home. Among children with three or fewer symptoms, rates of care-seeking from a trained provider remained below 8%. This rate more than doubled—to 18%—when four or more symptoms were present. The use of the village doctor was significantly higher for older children, and for illness perceived to be severe, or with danger signs, multiple symptoms, and longer duration. The presence of fever and diarrhoea also prompted greater care-seeking from the village doctors.

We observed a significant trend towards higher numbers of providers per episode among younger compared to older children. The perceived severi-ty, presence of at least one danger sign, number of symptoms, and duration of illness were all significantly associated with the number of providers from whom care was sought for an illness episode.

Table 3 presents the percentage of children seeking care from trained providers by sex, age, and household wealth quintile of the child's family, stratified by the perceived severity of the illness episode. The lack of association between sex of the child and care-seeking from a trained provider persisted across illnesses of varying severity. No associations were found between age of the child and the patterns of care-seeking (the association reported for mild episodes may be spurious as sample sizes for the youngest children were very small). For episodes perceived to be severe, children in the top two socioeconomic status quintiles were significantly more likely to seek care from a trained provider than children in the lower three quintiles. A similar but weaker pattern was observed for moderately-severe episodes.

**Table 3:** Percentage of sick children seeking care from trained providers by sex, age of child, socioeconomic status quintiles, stratified by perceived severity of illness episode

Sociodemographic characteristics	Perceived severity % (no. of sick children)		
	Mild	Moderate	Severe
Gender			
Boys	1.6 (125)	5.0 (342)	13.4 (247)
Girls	4.0 (151)	7.0 (287)	13.0 (184)
	p=0.30*	p=0.44	p=0.82
Age (months) of children			
0–5	15.0 (20)	5.4 (37)	14.3 (14)
6–11	0.0 (43)	8.7 (92)	20.7 (58)
12–23	5.9 (51)	4.2 (142)	15.1 (106)
24–35	1.7 (58)	5.0 (139)	8.8 (102)
36–47	1.7 (59)	4.8 (124)	9.9 (91)
48–59	0.0 (45)	8.4 (95)	15.0 (60)
	p=0.01*	p=0.76	p=0.20
Socioeconomic status			
Lo(l)	4.6 (44)	2.0 (147)	6.7 (90)
2	0.0 (61)	4.2 (118)	*7.7* (78)
3	4.6 (44)	6.4 (125)	11.6 (86)
4	1.6 (63)	5.6 (126)	16.1 (93)
Hi (5)	4.7 (64)	12.4 (113)	23.8 (84)
	p=0.354*	p=0.010	p=0.006

p values based on chi-square tests of independence/homogeneity comparing care from trained provider, other providers, and no care

*Exact tests comparing care from trained provider and others (other providers or no care)

### Home-care for common childhood illnesses

Home-care practices were investigated in three ways. First, we examined the proportions of ill children who received increased fluids and continued feeding throughout their illness, as recommended in the IMCI guidelines. Second, among children reported to have been taken to a trained provider, we examined the extent to which their caretakers reported that the recommendations of the provider had been adhered to at home. Third, among those children for whom providers recommended a follow-up visit, we examined the proportion for which the caretakers had either taken the child back to the facility or intended to do so within the recommended time period. Results for each of these sets of analyses are presented below.

Current home-care recommendations encouraged increased fluid and continued food intake for children during illness episodes. However, only 47% of the sick children in this sample were given at least the usual amount of fluids, about 34% continued to receive at least the usual amount of food, and only 4.0% received both increased fluids and continued feeding during the illness episode (data not shown). Children whose illness was perceived to be severe tended to be more likely to receive increased fluids and continued feeding compared to mild-to-moderate episodes (5.3% vs 2.9%; p=0.07). Illness with at least one danger sign also appeared to be more likely to be treated with increased fluids and continued feeding compared to episodes with no danger signs (5.4% vs 3.1%; p=0.10). There were no differences in this aspect of home-care either by sex of the child or by the socioeconomic status level of the household.

Table 4 shows the percentage of sick children seen by the trained providers whose caretakers reported that they either complied or intended to comply with the specific treatment recommended by the provider. The overall compliance was about 70%. However, the compliance was less common in children of the highest socioeconomic status and for illnesses of shorter durations, although the latter association did not reach a statistical significance.

**Table 4:** Percentage of sick children seen by trained providers and whose caretakers comply with treatment recommended by the provider

Sociodemographic and illness characteristics	Compliance with recommended treatment % (denominator)
Gender	
Boys	75 (57)
Girls	65 (51)
	p=0.22
Age (months) of children	
0–11	70 (27)
12–23	68 (28)
24–35	63 (19)
36–47	75 (16)
48–59	78 (18)
	p=0.88
Socioeconomic status	
Lo(l)	83 (12)
2	83 (12)
3	70 (20)
4	88 (25)
Hi (5)	51 (39)
	p=0.016†
Perceived severity of episode	
Mild	75 (8)
Moderate	65 (40)
Severe	73 (60)
	p=0.64
Presence of danger signs	
Yes	68 (44)
No	72 (64)
	p=0.68
Number of symptoms present*	
1	83 (18)
2	66 (38)
3	57 (23)
4 +	78 (18)
	p=0.28
Duration (days) of illness	
0–6	58 (33)
7–13	67 (27)
14 +	81 (48)
	p=0.06

p values based on chi-square tests of independence/homogeneity.

*Symptoms limited to: fever, cough, difficult/rapid breathing, diarrhoea, ear pain, generalized rash, sore throat, and eye problem (123 children did not have any of these symptoms and were not included in this analysis)

†Exact tests

We also asked caretakers who had sought care from a trained provider whether the provider had asked that the child be brought back for a follow-up visit. If a follow-up visit had been recommended, we asked whether they had complied, or intended to comply, with the recommendation. A follow-up visit had been recommended for only 39 children (36%) who were taken to a trained provider. Within this group, 41% of caretakers reported that they had either complied or intended to comply with the recommendation (data not shown). None of the factors under investigation (sex, age, socioeconomic status, perceived severity, presence of danger signs, number of symptoms, or duration of illness) was associated with the rate of compliance with follow-up, although small numbers make it impossible to have complete confidence in these results.

## DISCUSSION

This population-based sample survey provides new information about the morbidity patterns among Bangladeshi children and indicates that current rates of care-seeking and appropriate home-care must be improved in order for child mortality to continue to decline. Further analyses of these three intermediate outcomes by sex and age of the child, socioeconomic status of the household, and specific characteristics of the illness episode can help programme planners ensure that interventions reach all children who need them and inequities are minimized.

### Methodological issues in measuring socioeconomic status in rural Bangladesh

The validity of the asset index for measuring socioeconomic status may be challenged. The index was created using exactly the same methodology used by the World Bank group (10) to investigate differentials in country-level health indicators, but a slightly different set of variables had been adapted based on qualitative research in the local context. This approach to the measurement of inequalities has shown significant inequities in healthcare worldwide and has been widely used for influencing policy. The methodology itself is now widely used in this field. One direct source of confidence in validity of the asset index in this study is the presence of significant differentials in malnutrition by the asset index quintiles. This is very much what is expected and suggests that the index is indeed discriminatory.

At 41%, the prevalence of underweight among the top 20% of households is high and not much lower than the national average (48%) (2). In the Multicentre Growth Reference Study pilot survey in Dhaka, we had found the prevalence of underweight just below 5% in upper-income urban populations (unpublished results). Thus, ‘high socioeconomic status’ in Matlab still means being quite poor. The Matlab upazila study area should not be confused with the ICDDR,B intervention area in Matlab. Most health indicators in the ICDDR,B intervention area are much better than those in the rest of Matlab where this study was conducted (6).

### High morbidity and hospitalization rates, little evidence of inequities

The results showed a very high prevalence of illness among Bangladeshi children. Over two-thirds of the children were reported to have been ill during the two weeks preceding the survey. We compared the rates of reported morbidities in this survey with those reported for rural Bangladesh by the BDHS 1999–2000 (2). About 45% of the Matlab children had fever in the previous two weeks compared to 37% in the BDHS. Rates of diarrhoea were also higher in Matlab—13% compared to 6% reported nationally for rural Bangladesh. Matlab is known to have higher-than-average rates of diarrhoea (12), although there may also be seasonality-related differences between the two rates. It may be particularly noted that, unlike national rural rates of 19%, the two-week prevalence for probable pneumonia in our population was only about 4%. While the BDHS used a definition of cough with rapid breathing, we have used the MCE definition of cough and difficult or rapid breathing not due to blocked nose. However, if we use the less demanding of definition of cough and difficult or rapid breathing, the prevalence is 13%. Seasonality may explain the difference in pneumonia rates between the two surveys, as the BDHS was carried out in the winter months of 1999–2000 when rates of respiratory illnesses are usually higher, and our survey was carried out in the warmer months of April–October and over a longer period.

An important rationale for the development of the IMCI strategy was the belief that sick children often present with more than one symptom. This management of the child in a holistic approach has not been a strong point of disease-specific vertical programmes. Our data showed that most sick children in this population had multiple symptoms, suggesting that the use of the IMCI clinical management guidelines will lead to improved quality of care.

Contrary to expectations, there were no significant differences in the prevalence of illness by sex or by socioeconomic status, although we did observe higher rates of perceived severe illness in boys which may be indicative of a bias in recognition of illness. The lack of a socioeconomic status difference needs to be treated with some caution because these were reported morbidities that can be affected by recognition of illness and adequacy of recall, both of which are likely to be better in higher socioeconomic status groups. These biases may have masked effects of socioeconomic status on the prevalence of illness.

We were surprised by the low rates of hospitalization in our study population and by the lack of clear differences by economic status, despite a non-significant trend towards higher rates among those with more resources. It is possible that variations by economic status were not apparent because of small numbers, but the overall low rate of hospitalization is difficult to explain given that there are two hospitals serving the area, including one specialized diarrhoea hospital. Reports from other countries would lead us to expect higher rates of hospitalization (13). Evidence from formative research in the study area suggests that the low rates found in this setting can be explained by a combination of factors, including inherent dislike of hospitalization, poor perceived quality of services, low perceived need (relates to perception of the illness, and perceived apppropriate care), direct and indirect costs, concerns about family responsibilities while being hospitalized, and concerns about transport and stay at the hospital may all contribute to the low rates of hospitalization (Blum L. Personal communication, 2004). Concerning the economic differentials, it is possible that wealthier families have less morbidity which is then masked by improved recognition, recall, and care-seeking. In Tanzania, those with more resources are hospitalized significantly more often than those with fewer resources, and there are few hospitalizations among those in the poorest quintiles (14). In Brazil where over 10% of under-five children are hospitalized in any given year, the poor are hospitalized more often than the rich (15).

### Differentials in care-seeking by socioeconomic status

Poorer children were less likely to be taken to an appropriate healthcare provider than less-poor children, even in this apparently homogeneous and very poor rural Bangladeshi population. Our findings indicated that children with severe illness in the least poor households were three times more likely to seek care from a trained provider than children in the poorest households. Gwatkin and his colleagues found similar socioeconomic status differentials while analyzing data from the DHS 1996–1997 (10). Gender inequities in child healthcare had been previously reported from Bangladesh (16,17), but were not found in this survey in terms of the prevalence of illness (as reported above) or care-seeking patterns, although hospitalization rates were significantly higher (66%) among boys. Our findings are consistent with recent evidence of narrowing gender differentials in child mortality and other healthcare indicators in Bangladesh (18).

Although the Government of Bangladesh has an excellent public-health infrastructure—at least one primary care facility for population of 20,000 and a 31-bed hospital for 200,000 people—care-seeking for childhood illnesses, particularly from the formal health sector, was extremely low. About one-third of children with a reported illness did not receive any care outside the home. The majority of sick children for whom care was sought outside the home were taken to a village doctor. Only 8% went to a trained provider, i.e. a health facility, a hospital, a doctor, paramedic, or a community health worker. This rate of use of a trained provider is unexpectedly low. In the BDHS 1999–2000, 27% of children with probable pneumonia were taken to a health facility (2). Based on the MCE definition of cough and difficult or rapid breathing not due to blocked nose, our survey found that only 7% of these children with probable pneumonia were taken to trained providers. However, if we use the broader definition of cough and difficult or rapid breathing (without excluding children who have a blocked nose), care-seeking from trained providers increases to 12%. In a recent national ARI/CDD survey conducted by the Government, caretakers of 58% of children with fast or difficult breathing were taken outside the home for care, and 22% sought care from the formal health sector (19).

The low level of care-seeking from the formal health sector documented in this analysis is of concern and provides further consistent evidence that children are not currently benefiting from efforts to improve the quality of care delivered by the health system. This presents particular challenges for the implementation of IMCI, for which facili-ty-based services have usually been implemented more strongly than community interventions (20). At such low levels of use, it seems unlikely that IMCI will have a major impact on child mortality unless care-seeking behaviours improve. The perceived quality of care was found to be one of the important factors in healthcare-seeking behaviour (21), which provides hope in this context as the implementation of IMCI is expected to improve the quality of care at the health facility.

Our results indicated that care-seeking was influenced by the perceived severity of the illness, presence of danger signs, and duration and number of symptoms. These are positive findings that can be used as the basis for designing effective behaviour-change interventions. We will need to overcome the sex differential in perception of severity as it is mainly through the management of severe illness that we expect to see a mortality impact. Perceptions and beliefs surrounding childhood illness and healthcare providers clearly influence care-seeking. A study in the early 1990s examined these issues in the Matlab area (22). In that study, although mothers recognized illness, care-seeking and choice of providers were influenced by a multitude of factors, including perceived disease-causation mechanisms, access to and costs of care, and quality of care.

The majority of the estimated 10 million deaths that occur each year in developing country among under-five children can be prevented or the illness be effectively treated by available interventions (23). However, case-management interventions require a high level of appropriate care-seeking for illness. It will be important to identify the reasons for such infrequent use of trained providers, despite the establishment of an extensive public-health infrastructure in Bangladesh. There is abundant evidence in the literature of an association betweem perceived quality of care and care-seeking. The decision of wherther or not to take the child to a health centre depended on the child's condition, the availablity of drugs, and perception of the quality of care offered at health centres (21,24). It is possible that the low use can largely be attributed to non-functioning of the GoB facilities and/or to poor quality of care provided by these facilities (25). The observed sex differential in hospitalization will also need to be addressed.

Since untrained providers are the major source of care (26), they can be a potential target for strate-gies to improve child survival (27). Bringing on board untrained providers might lead to an understanding of how they can help identify severely-ill children and refer them to facilities able to provide appropriate treatment.

### Caretakers are responding to advice from healthcare providers

Compliance with the advice given was reasonably high among those who sought care from trained providers. This suggests that IMCI facility-based interventions will be highly effective in reducing child mortality if high-enough levels of use could be achieved. However, the high levels of compliance reported here could also be an artifact because mothers were asked whether they complied with their own recollection of the instructions of the health workers rather than with what the health workers actually told them to do.

The results indicating higher rates of compliance for children living in poorer households is puzzling, although we also observed that the poor also performed equally well regarding home-care. In the absence of previous research on this topic, we speculate that those with higher levels of resources are more willing to use those resources to seek care from multiple sources and are, thus, less likely to comply with a particular provider's advice, or that they may be more willing to express their intent not to comply with. An examination of our data actually showed that those in the highest socioeconomic status quintile sought care from more providers for the same episode, although the differences were not significant. Qualitative research is needed to investigate these issues. In the meantime, interventions that require compliance with medical advice may be more effective among poorer children and could lead to reductions in the differentials found for care-seeking.

This population-based sample survey using standardized instruments adapted for Bangladesh was conducted in the context of a large intervention study which was carried out in collaboration with the Government. The results highlight the challenges that will need to be addressed as IMCI or similar maternal and child health programmes are implemented in health facilities and extended to address key family and community practices.

The most daunting challenge is the extremely low rates of use of the formal health sector for the management of sick children. Child health planners must find ways to address the apparent population preference for untrained and traditional providers, the differentials in care-seeking practices that discriminate against the poorest households, and the sex differentials in the perception of severity and hospitalization.

Efforts similar to those reported here can be used as formative research for the continued improvement of child health services. Future reports on the implementation of IMCI in Matlab upazila as part of the global Multi-country evaluation can provide timely evidence of progress and identify areas for improvement in advance of the full-scale implementation of IMCI planned by the Government. Research along the lines presented here, complemented by qualitative studies aimed at understanding the determinants of care-seeking behaviours, is essential to provide a solid basis for strong programme planning and implementation.

## ACKNOWLEDGEMENTS

This research study was conducted at ICDDR,B with support from the Bill and Melinda Gates Foundation through a grant to the Department of Child and Adolescent Health and Development of WHO and Cooperative Agreement No. 388-A-00-97-00032-00 from the Bangladesh Mission of the United States Agency for International Development. ICDDR,B acknowledges with gratitude the commitment of WHO, the Gates Foundation, and USAID to the Centre's research effort.

This work is a part of the Multi-country Evaluation (MCE) of IMCI Effectiveness, Cost and Impact. This work could not have been done without the full cooperation of the families in the survey area. The authors appreciate their willingness to be interviewed, especially prior to the introduction of IMCI. The authors would also like to thank the MCE Central Team for their continued technical and administrative support to the study and the MCE Technical Advisors for important suggestions on earlier drafts of this paper and continued guidance through the course of the study.

Finally, the authors deeply thank the partners in the Government of Bangladesh. The authors note their commitment to this study and for using the results of the research to improve child health plans and policies in Bangladesh.
